# Incidental hepatocellular carcinoma after liver transplantation: Prevalence, histopathological features and prognostic impact

**DOI:** 10.1371/journal.pone.0175010

**Published:** 2017-04-12

**Authors:** Pablo Pérez, Manuel Rodríguez-Perálvarez, Lourdes Guerrero, Víctor González, Rafael Sánchez, Macarena Centeno, Antonio Poyato, Javier Briceño, Marina Sánchez-Frías, Jose Luis Montero, Manuel De la Mata

**Affiliations:** 1 Department of Hepatology and Liver Transplantation, Reina Sofía University Hospital, Córdoba, Spain; 2 Maimónides Institute of Biomedical Research of Córdoba (IMIBIC), Córdoba, Spain; 3 CIBERehd, Instituto de Salud Carlos III, Madrid, Spain; 4 Department of Pathology, Reina Sofía University Hospital, Córdoba, Spain; University of North Carolina at Chapel Hill School of Medicine, UNITED STATES

## Abstract

**Background:**

Incidental hepatocellular carcinoma (iHCC) is a histological finding after liver transplantation (LT) which relevance has been scarcely studied.

**Aims:**

to describe the histopathological features of iHCC and to determine its prognostic impact in terms of tumor recurrence and overall survival.

**Methods:**

Observational study including 451 consecutive adult LT patients (2000–2013). Patients aged<18, retransplanted or with early postoperative death were excluded. Median follow-up after LT was 58 months. Multiple Cox’s regression was used to assess the prognostic impact of iHCC on tumor recurrence and mortality while controlling for potential confounders.

**Results:**

141 patients had known HCC before LT (31.3%). Among the remaining 310 patients, the prevalence of iHCC was 8.7% (n = 27). In the explanted liver, 36.2% of patients with known HCC and 25.9% of patients with iHCC trespassed Milan criteria (*p* = 0.30). Patients with known and iHCC had similar rates of multinodular disease (50.4% vs 55.6%; *p* = 0.62), macrovascular invasion (6.5% vs 3.7%; *p* = 0.58), microvascular invasion (12.9% vs 14.8%; *p* = 0.76) and moderate-poor tumor differentiation (53.9% vs 70.4%; *p* = 0.09). In the multivariate analysis, iHCC and known HCC had identical recurrence-free survival after controlling for histological features (RR = 1.06, 95%CI 0.36–3.14; *p* = 0.90). Cumulative 5-year overall survival rates were similar between patients with known and iHCC (65% vs 52.8% respectively; log rank *p* = 0.44), but significantly inferior as compared with patients without HCC (77.8%) (*p* = 0.002 and *p* = 0.007 respectively). Indeed, in the overall cohort, iHCC was an independent predictor of mortality (RR = 3.02; 95%CI 1.62–5.65; *p* = 0.001).

**Conclusion:**

The risk of tumor recurrence after LT is similar in patients with iHCC and known HCC. A close imaging surveillance is strongly recommended for patients awaiting LT in order to detect HCC prior to LT, thus allowing for an adequate selection of candidates, prioritization and indication of bridging therapies.

## Introduction

Hepatocellular carcinoma (HCC) is the main cause of death among patients with end-stage liver disease, and the second leading indication for liver transplantation (LT) in Europe and US [1, 2]. Indeed, LT is the best therapeutic option for cirrhotic patients with HCC who meet Milan criteria [3] (ie. one nodule less than 5 cm, or up to three nodules less than 3 cm each, in absence of macrovascular invasion or extrahepatic spreading). However, HCC recurrence may occur in up to 20% of patients, and even more frequently in patients above Milan criteria [4]. The key strategies to prevent tumour progression and derived drop-out, while minimizing the risk of tumor recurrence after LT, include an adequate selection of candidates for LT, an optimized prioritization within the waiting list, and an appropriate indication of neoadjuvant locoregional therapies [5, 6].

The characterization of small focal lesions in cirrhotic livers is challenging, especially for those tumors smaller than 2 cm [7]. In addition, an advanced HCC with a diffuse pattern may not be detected in patients with a heterogeneous liver parenchyma [8, 9]. It has been suggested that an intensive imaging surveillance by using high resolution dynamic techniques (ie. Computed tomography (CT) and/or magnetic resonance imaging (MRI)) protocolized in short intervals would allow to refine the diagnostic algorithm [10]. However, despite the technologic development observed in recent years, HCC remains elusive in some patients before LT, and it is sometimes unexpectedly diagnosed in the histopathologic examination of the explanted liver, in what has been termed as “incidental hepatocellular carcinoma” (iHCC). This subset of patients with iHCC may not benefit from any of the above referred strategies to prevent tumor recurrence [11]. The prevalence of iHCC varies widely among the available reports (4.2% to 40%) [10, 12–19], and it is unclear whether iHCC has a detrimental prognostic impact when compared with patients with previously known hepatocellular carcinoma (kHCC). The number of studies is limited, and most of the evidence comes from small non-controlled series with contradictory findings [10, 12–19].

The aims of the present study were to describe the pathological features of iHCC, and to determine its prognostic impact in terms of tumor recurrence (as compared with kHCC) and overall survival (as compared with patients with kHCC and without HCC).

## Methods

The present clinical investigation has been conducted according to the principles contained in the Declaration of Helsinki. None of the transplant donors were from a vulnerable population and all donors or next of kin provided written informed consent that was freely given. This project is part of a broader research initiative (PI14/01469), which was approved by our local Ethics Committee (Comité de Ética de la Investigación de Córdoba) on 30th March 2015.

This retrospective observational study included a cohort of 663 consecutive patients who underwent LT from January 2000 to April 2013 at a single institution. Exclusion criteria were: Age <18 years old, retransplantation and early postoperative death (ie. within the first month). Two hundred and twelve patients were excluded according to these criteria, and thus the final study population comprised 451 patients. The screening of HCC among patients with cirrhosis was performed by using liver ultrasound every 6 months according to international guidelines [7]. Computed tomography and/or magnetic resonance imaging were ordered if the patient had a suspicious liver nodule detected in the liver ultrasound or showed raising alpha-fetoprotein otherwise unexplained. Patients with HCC were considered eligible for LT provided that they fulfilled Milan criteria. Once the patient was included in the waiting list, a liver imaging technique (ultrasound, computed tomography or magnetic resonance) was performed every 3 months for all patients irrespective of the existence of prior diagnosis of HCC. Those patients with HCC experiencing tumor progression beyond Milan criteria were removed from the waiting list. Locoregional ablative therapies were indicated prior to LT unless technically unfeasible. The preferred locoregional ablative therapy was transarterial chemoembolization. Prioritization within the waiting list in patients without evidence of HCC was performed by using the MELD score. Patients with kHCC prior to LT entered the waiting list with a MELD score of 15, and received 1 extra MELD point in a monthly basis if they had one nodule >3cm or multinodular disease.

The explanted liver was examined by an experienced liver pathologist, who provided a detailed description of the number of nodules and size of the HCC, along with the presence of satellite nodules or capsule invasion. All suspicious nodules detected in the macroscopic examination were microscopically reviewed. Tumor differentiation was graded according to Edmonson’s scale [20]. Microvascular invasion was defined either as a tumor emboli within a peritumoral vessel, or as a satellite nodule surrounded by endothelium (positive for CD34 staining) [21]. iHCC was diagnosed in the explanted liver by histological examination, provided that no HCC was apparent in the pre-transplant imaging assessment [7].

Primary immunosuppression consisted in tacrolimus, mycophenolate and tapering corticosteroids for most patients. Tacrolimus trough concentrations were kept between 7–10 ng/mL within the first month after LT, and 4–8 ng/mL thereafter, as this protocol has been associated with prolonged graft survival [22] and reduced HCC recurrence rates [23]. Patients with pre-transplant renal dysfunction or perioperative hepatic encephalopathy received induction therapy with basiliximab (on post-operative days 1 and 5) and delayed tacrolimus initiation (at day 5–7 post-LT). From 2012, patients with HCC (both incidental and previously known) received reduced tacrolimus and early initiation of everolimus as part of an observational study [24]. The median follow-up after LT was 58 months (IQR 29–112) for the whole cohort. Patients with HCC (iHCC and kHCC) underwent liver ultrasound and serum alpha-fetoprotein every 3 months within the first post-transplant year, and every 6 months thereafter to detect tumor recurrence.

### Statistical analysis

Statistical analysis was performed by using SPSS^®^ software version 15 (IBM Corp., Chicago, USA). Variables were displayed in frequency tables or expressed as means and standard deviations, except for those with asymmetric distribution in which median and interquartile range were used. Chi square test was used for frequencies, Student's t test or ANOVA for continuous variables, and Mann—Whitney's U or Kruskal—Wallis for asymmetric distributions. *P* <0.05 was considered significant. Kaplan—Meier curves (Log-rank test) and multiple Cox’s regression analysis were used to explore the impact of iHCC on overall survival and recurrence free survival. The initial multivariate Cox’ model was composed by those variables with a *p* <0.20 in the univariate analysis. The elimination of covariates was performed in a stepwise backward process. All possible interactions among variables were tested. Variables with a *p* value between 0.05 and 0.15 were screened to identify potential confounding factors, and further removed from the model if they did not behave as such.

## Results

### Patients baseline and clinical characteristics

A total of 451 consecutive adult LT patients were included. In 141 patients (31.3%) the diagnosis of HCC was performed prior to LT (ie. kHCC group). Among the remaining 310 patients without HCC according to the pre-LT imaging assessment, 27 patients showed an iHCC in the histological examination of the explanted liver (prevalence 8.7%). In the whole cohort the mean age was 61.1 ± 10.1 years and there was a male predominance (n = 340; 75.4%). The most frequent aetiologies of liver disease were: alcoholic liver disease (n = 196; 43.5%), chronic hepatitis C (n = 191; 42.4%) and chronic hepatitis B (n = 53; 11.8%). The median length within the waiting list was 5 months (IQR 1.4–9.3). Mean donor age was 49.3 ± 17.5 years.

The baseline characteristics of the included patients are summarized in Table 1. Patients with iHCC were younger than patients with kHCC (60.7 ± 7.2 versus 64.8 ± 7.6 years respectively; *p* = 0.01). There were no differences in terms of gender distribution. Aetiology of liver disease was homogeneous across groups, except for chronic hepatitis C, which was predominant in patients with iHCC, as compared with the remaining cohort (63% vs 36%; *p* = 0.006). Patients with iHCC had increased Child-Pugh score compared with kHCC patients (*p* <0.01). Similarly, complications derived from portal hypertension were more prevalent in patients with iHCC as compared with kHCC (96.3% vs 75.2%; *p* = 0.006). Patients with iHCC had ascites, hepatic encephalopathy and hepatorenal syndrome more frequently than patients with kHCC (*p* <0.01, *p* <0.01 and *p* = 0.016 respectively). Length within the waiting list and follow-up after LT were homogeneous among kHCC and iHCC groups (6.02 ± 5.41 vs 7.19 ± 9.54 months, *p* = 0.373; and 63.04 ± 46.3 vs 62.81 ± 52.74 months, *p* = 0.982, respectively). Pretransplant alpha-fetoprotein serum concentration was significantly lower in patients with iHCC (3.94 ng/dL; IQR 1.93–23.75) than in patients with kHCC (11 ng/dL; IQR 5.1–45.5) (*p* = 0.008). In patients with kHCC, the initial diagnosis was made by using magnetic resonance imaging (46.1%), computed tomography (31.9%) or liver ultrasound (22%). Eighty-six patients (61%) with kHCC underwent neoadyuvant locoregional therapies before LT, being transarterial chemoembolization the most frequently indicated procedure (43.9% of patients).

**Table 1:** Clinical characteristics of 451 consecutive patients who received a liver transplantation between January 2000 to April 2013. Patients were stratified into previously known hepatocellular carcinoma (kHCC, n = 141), incidental hepatocellular carcinoma (iHCC, n = 27), and patients without hepatocellular carcinoma (nHCC, n = 283). Continuous variables are presented as mean ± standard deviation (median and interquartile range for asymmetric distributions). Categorical variables are displayed as n (%). Statistically significant findings are highlighted in bold.

Variable	kHCC	iHCC	nHCC	*p* value	*p* value
	(n = 141)	(n = 27)	(n = 283)	(iHCC vs kHCC)	(iHCC vs nHCC)
**Age at LT (years)**	**64.8 (± 7.6)**	**60.7 (± 7.2)**	59.4 (± 10.9)	**0.01**	0.508
**Gender, n (%):**					
• **Male** • **Female**	• 126 (89.4%) • 15 (10.6%)	• 22 (81.5%) • 5 (18.5%)	• 192 (67.8%) • 91 (32.2%)	0.247	0.143
**Aetiology of liver disease, n (%):**					
• **Alcohol** • **HCV** • **HBV** • **Other**	• 64 (45.4%) • 72 (51.1%) • 28 (19.9%) • 3 (2.1%)	• 13 (48.1%) • **17 (63%)** • 2 (7.4%) • **1 (3.7%)**	• 119 (42%) • **101 (36%)** • 23 (8.1%) • **61 (21.6%)**	• 0.792 • 0.256 • 0.171 • 0.507	0.54 **0.006** 0.896 **0.023**
**Child-Pugh liver score, n (%):**					
• **A** • **B** • **C** • **Missing values**	• **90 (63.8%)** • **29 (20.6%)** • **(2.8%)** • **18 (12.8%)**	• **1 (3.7%)** • **15 (55.6%)** • **8 (29.6%)** • **3 (11.1%)**	• 42 (14.8%) • 127 (44.9%) • 69 (24.4%) • 45 (15.9%)	**<0.001**	0.236
**Portal hypertension, n (%):**	**106 (75.2%)**	**26 (96.3%)**	**233 (82.3%)**	**0.006**	**0.002**
**Previous decompensations, n (%):**					
• **Ascites** • **HE** • **VGB** • **SBP** • **HRS**	• **54 (38.3%)** • ** 21 (14.9%)** • 18 (12.8%) • (4.3%) • **3 (2.1%)**	• **26 (96.3%)** • **20 (74.1%)** • (22.2%) • 4 (14.8%) • **4 (14.8%)**	• **202 (71.4%)** • **128 (45.2%)** • 62 (21.9%) • 32 (11.3%) • 25 (8.8%)	• **<0.001** • **<0.001** • 0.49 • 0.069 • **0.016**	**0.009** **0.03** 0.888 0.757 0.324
**Length within waiting list (months)**	6.02 (± 5.41)	7.19 (± 9.54)	7.74 (± 9.05)	0.373	0.764
**Follow-up after LT (months)**	63.04 (± 46.3)	62.81 ± (52.74)	72.98 (±52.85)	0.982	0.34

**Abbreviations:** kHCC, previously known hepatocellular carcinoma; iHCC incidental hepatocellular carcinoma; nHCC neither previously known nor incidental hepatocellular carcinoma; LT, liver transplantation; HCV, hepatitis C virus; HBV, hepatitis B virus; HE, hepatic encephalopathy; VGB, variceal gastrointestinal bleeding; SBP, spontaneous bacterial peritonitis; HRS, hepatorenal syndrome.

### Histopathological features of iHCC as compared with kHCC

The histopathological features of patients with iHCC and kHCC are displayed in Table 2. Among patients with kHCC, the prevalence of multinodular disease was 50.4%, as compared with 55.6% of patients with iHCC (*p* = 0.621). There was no difference in the diameter of the main nodule between kHCC (median 3 cm; IQR 2–4) and iHCC (median 1.55 cm; IQR 1.2–2.5) (*p* = 0.369). However, total tumor volume (ie. sum of the diameter of all identified nodules) was marginally reduced in patients with iHCC (median 2.35 cm; IQR 1.5–3.75), as compared with patients with kHCC (median 3.50 cm; IQR 2.5–5.5) (*p* = 0.046). In terms of tumor differentiation, no statistical differences were found between kHCC and iHCC (*p* = 0.094), although this information was not available in 21 patients with kHCC who showed a massive tumor necrosis derived from pre-LT locoregional ablative therapies. The rates of macrovascular invasion and microvacular invasion were similar in patients with iHCC and kHCC (6.5% vs 3.7%, *p* = 0.58; and 12.9% vs 14.8%, *p* = 0.76 respectively). In the explanted liver, 51 patients with kHCC (36.2%), and 7 patients with iHCC (25.9%) were beyond Milan criteria (*p* = 0.30).

**Table 2:** Histological features of hepatocellullar carcinoma in the explanted liver. Patients were stratified into previously known hepatocellular carcinoma (kHCC, n = 141) and incidental hepatocellular carcinoma (iHCC, n = 27). Descriptive values are displayed as median (interquartile range) or N (%). Statistically significant findings are highlighted in bold.

Variable	kHCC	iHCC	*p* value
	(n = 141)	(n = 27)	
**Diagnostic imaging technique, n (%):**			
**Ultrasound** **CT** **MRI**	13 (9.2%) 45 (31.9%) 65 (46.1%)		
**Pre-LT AFP (ng/dL): median (IQR)**	**11 (5.1–45.5)**	**3.94 (1.93–23.75)**	**0.008**
**Locoregional therapy pre-LT**	86 (61%)	0 (0%)	—
**Number of nodules, n (%):**			
**Uninodular** **Multinodular**	69 (49.6%) 70 (50.4%)	12 (44.4%) 15 (55.6%)	0.62
**Main nodule diameter (cm): median (IQR)**	3 (2–4)	1.55 (1.2–2.5)	0.37
**Total tumor size (cm): median (IQR)**	**3.50 (2.5–5.5)**	**2.35 (1.5–3.75)**	**0.046**
**Tumor differentiation:**			
**Well** **ModeratePoor** **Unavailable data**	41 (29.1%) 65 (46.1%) 11 (7.8%) 21 (14.9%)	8 (29.6%) 18 (66.7%) 1 (3.7%) 0 (0%)	0.09
**Macrovascular invasion, n (%)**	9 (6.5%)	1 (3.7%)	0.58
**Microvascular invasion, n (%)**	18 (12.9%)	4 (14.8%)	0.76
**Within Milan criteria, n (%)**	90 (63.8%)	20 (74.1%)	0.30

**Abbreviations:** kHCC, previously known hepatocellular carcinoma; iHCC incidental hepatocellular carcinoma; CT, computed tomography; MRI, magnetic resonance imaging; LT, liver transplantation; AFP, alpha-fetoprotein; IQR, interquartile range.

### Predictors of overall survival and recurrence-free survival

**Fig 1:**
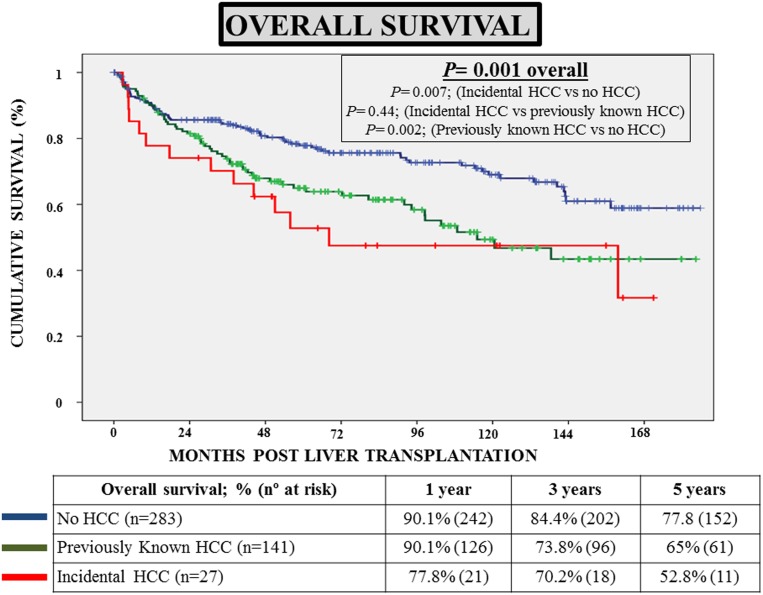
Survival curves showing overall survival of patients with incidental hepatocellular carcinoma (n = 27), as compared with patients with previously known hepatocellular carcinoma (n = 141), and patients without hepatocellular carcinoma (n = 283).

The median follow-up after LT was 58 months (IQR 29–112). Overall survival rates were similar between patients with iHCC (70.2% at 3 years and 52.8% at 5 years) and patients with kHCC (73.8% at 3 years and 65% at 5 years) (*p* = 0.44), but significantly lower than those exhibited by patients without HCC (84.4% at 3 years and 77.8% at 5 years) (*p*< 0.01 for both comparisons) (Fig 1). Multivariate Cox’s regression was used to identify independent predictors of overall survival after LT. The initial model was composed by the following variables: age, gender, pre-LT MELD, aetiology of liver disease (hepatitis C), aetiology of liver disease (alcoholic liver disease), length within the waiting list and HCC status (iHCC vs kHCC vs nHCC). The following covariates were removed one by one from the model because of their lack of significance (*p*> 0.15): gender (*p* = 0.95), pre-LT MELD (*p* = 0.85), length within the waiting list (*p* = 0.74) and alcoholic liver disease (*p* = 0.58). A significant interaction was found between aetiology of liver disease (hepatitis C) and HCC status, and therefore was kept in the model. The final model is summarized in Table 3. The independent predictors of mortality were: age of the recipient (*p* = 0.007), presence of HCC (*p* = 0.001), and the interaction between HCC and hepatitis C status (*p* = 0.001). It is noteworthy that, among patients with HCC, only the subset of patients with iHCC had a significant detrimental impact on overall survival (RR = 3.02; 95%CI 1.62–5.65; *p* = 0.001).

**Table 3:** Multivariate Cox’s regression showing independent predictors of overall survival after liver transplantation.

	Wald	gl	Sig.	RR	95% CI for RR
1. Age of the recipient	7.271	1	0.007	1.029	[1.008, 1.051]
2. HCC status		2	<0.01		
nHCC vs iHCC kHCC vs iHCC	12.109 1.391	1 1	<0.01 0.24	0.331 0.675	[0.177, 0.617] [0.315, 1.297]
3. Interaction HCC status/hepatitis C	13.063	2	<0.01		
kHCC vs iHCC/hepatitis C nHCC vs iHCC/hepatitis C	12.986 0.075	1 1	<0.01 0.78	2.336 1.075	[1.473, 3.707] [0.642, 1.799]

**Abbreviations:** RR, relative risk; CI, confidence interval; HCC, hepatocellular carcinoma; nHCC neither previously known nor incidental hepatocellular carcinoma; iHCC incidental hepatocellular carcinoma; kHCC, previously known hepatocellular carcinoma.

**Fig 2:**
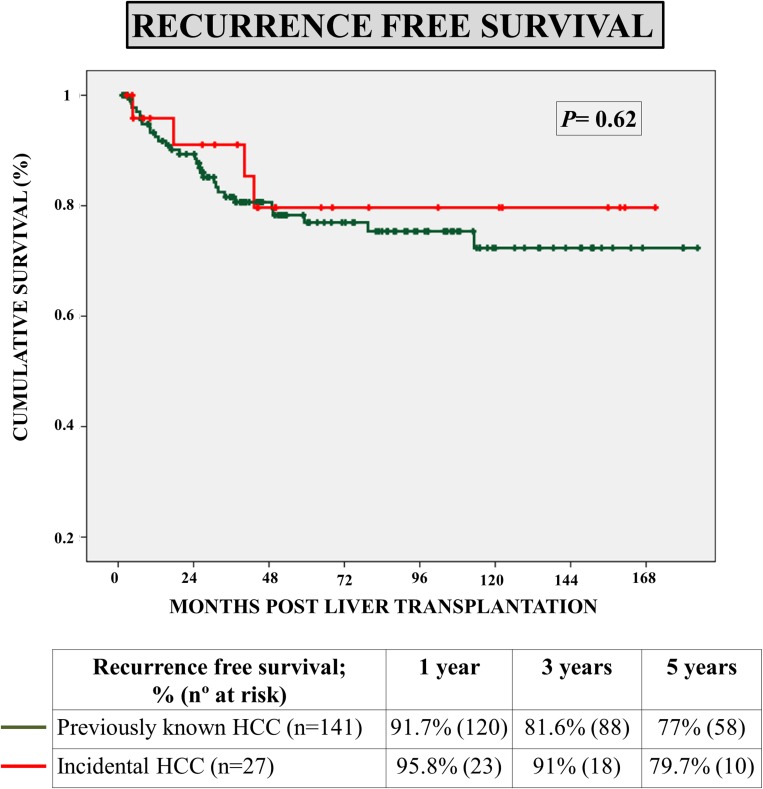
Survival curves showing recurrence-free survival of patients with incidental hepatocellular carcinoma (n = 27), as compared with patients with previously known hepatocellular carcinoma (n = 141).

Recurrence-free survival rates at 3 and 5 years post-LT were 91% and 79.7% respectively in patients with iHCC, and 81.6% and 77% respectively in patients with kHCC, without statistical significance (*p* = 0.62) (Fig 2). Again, a multivariate Cox’s regression model was designed to investigate the independent predictors of recurrence-free survival. The initial model comprised the following covariates: HCC status (iHCC vs kHCC), length within waiting list, pre-LT alpha-fetoprotein, aetiology of liver disease (hepatitis C), Milan criteria fulfillment, microvascular invasion and tumor differentiation. One by one, the following not significant variables were removed: chronic hepatitis C (*p* = 0.96), length within the waiting list (*p* = 0.58), alpha-fetoprotein (*p* = 0.21) and HCC status (*p* = 0.90). No significant interaction was found among the remaining covariates. Grade of tumor differentiation had a *p* = 0.13, and was further removed, as soon as its role as confounding factor was ruled out. The only independent predictors of tumor recurrence were microvascular invasion (RR = 5.3; 95%CI 2.57–11.08; *p*<0.01) and tumor beyond Milan criteria in the explanted liver (RR = 4.39; 95%CI 2.08–9.26) (Table 4). As noted above, iHCC patients had a comparable risk of tumor recurrence as patients with kHCC (RR = 1.06, 95%CI 0.36–3.14; *p* = 0.90), after controlling for Milan criteria fulfillment, microvascular invasion and grade of tumor differentiation.

**Table 4:** Multivariate Cox’s regression showing independent predictors of recurrence-free survival after liver transplantation.

	Wald	gl	Sig.	RR	95% CI for RR
Beyond Milan criteria	15.105	1	<0.01	4.39	[2.082, 9.256]
Microvascular invasion	20.141	1	<0.01	5.334	[2.568, 11.080]
Differentiation grade	2.244	1	0.13	1.932	[0.816, 4.575]

**Abbreviations:** RR, relative risk; CI, confidence interval.

## Discussion

LT is the only therapeutic option able to cure both, liver cirrhosis and HCC. Tumor recurrence rates are still 15%-20%, but may be minimized by an adequate selection of candidates for LT, indication of pre-LT locoregional ablative therapies, and appropriate prioritization within the waiting list [6]. In this setting, iHCC poses a great challenge since it does not allow for any of the previously referred strategies. The present study showed that iHCC is frequent and is associated with a significant risk of tumor recurrence, which is comparable to that observed in patients with kHCC, but with an even more pronounced impact on overall survival.

The heterogeneity of the liver parenchyma may experience a parallel increase with the progression of the liver disease. In patients with end stage liver disease the rates of misdiagnosis of small liver nodules by using liver ultrasound increases exponentially. In the present study, iHCC patients were older, had worsened liver function, and more complications derived from portal hypertension than patients undergoing LT without HCC. These findings are in line with previous reports [12, 13, 16, 19, 25]. In addition, patients with iHCC had more frequently chronic hepatitis C, as previously shown in some series [12, 15]. The pathogenesis of HCC is characterized by maintained inflammation, fibrosis and regeneration, mechanisms particularly prominent in chronic viral hepatitis. Thus, this subgroup of patients with hepatitis C and advanced cirrhosis should be included into specific surveillance protocols within the waiting list, including more frequent high resolution imaging techniques (ie. CT and/or MRI) and biopsy of any suspicious nodule detected.

Some clinical reports have suggested that patients with iHCC usually have small uninodular lesions, being the risk of derived tumor recurrence negligible [13, 17]. Nevertheless, our results challenge this statement. Patients with iHCC often had poor prognostic histological features and were found beyond Milan criteria in the explanted liver. Microvascular invasion seems to be the most aggressive histological feature, able to triple the risk of tumor recurrence after LT [21]. Since there is no consensus on the optimal histopathological examination protocol of the explanted liver, a significant disagreement among pathologists would be expected concerning the diagnosis of small uninodular incidental lesions. It is noteworthy that, among previous reports, the prevalence of iHCC inversely correlated with the rates of microvascular invasion and tumor recurrence, suggesting a more detailed examination of the explanted liver in studies with increased prevalence of iHCC and earlier tumor stages. Therefore, the more prevalent iHCC is, the less impact in terms of tumor recurrence may be expected. For instance, in the study by Piñero et al, 28% of the HCC patients had iHCC, and only 7% of these experienced tumor recurrence at 5 years[16]. In contrast, another report showing a reduced prevalence of iHCC (ie. 16.6% of the whole HCC transplanted population), the risk of tumor recurrence was at 5 years was 4-fold increased (ie. 30%) [17]. This forms a major source of bias, which could only be avoided by controlling histopathological features of HCC as potential confounding factors. This is the first study in which multivariate Cox regression was used to control for microvascular invasion status, tumor differentiation and Milan criteria fulfilment, and therefore provides more solid evidence about the true clinical relevance of iHCC in LT. A similar risk of HCC recurrence was found between patients with iHCC and kHCC, suggesting that the earlier stage at transplant in some cases with iHCC may be counteracted by the inadequate selection and prioritization of candidates, and by the absence of bridging locoregional therapies. Since the former strategy may prevent very aggressive HCCs to be transplanted [26], and the later would hinder tumor progression within the waiting list [11, 27], an active screening may be considered to diagnose HCC while the patient is still on the waiting list.

Therapeutic options are scarce once HCC recurrence has occurred, being this complication associated with premature death. The actual clinical relevance of iHCC should be quantified in terms of excess of tumor recurrence and negative impact on overall survival within the whole transplant population in order to preserve the utility-based principle. It is widely accepted that the rates of tumor recurrence in patients transplanted above Milan criteria are too high, forming a significant handicap for patients accessing the waiting list with other indications. A moderate expansion of Milan criteria by using the “up-to-seven” criteria (ie. Sum of the diameter of the largest nodule and the number of nodules less than 7) did not increase tumor recurrence rates, provided that microvascular invasion was absent [4]. In our cohort, 25% of patients with iHCC were above the “up-to-seven” criteria, or trespassed Milan criteria but had microvascular invasion. This subset of patients, if diagnosed preoperatively, should not have been transplanted according to international guidelines [7]. Instead, they would have received alternative therapies, resulting in reduced morbidity derived from LT, and in a benefit for the waiting list population. Although there were no significant differences between iHCC and kHCC in terms of disease free and overall survival, aligning with previous observations [13, 17, 28], a trend to worse survival was observed in iHCC patients, which did not reach statistical significance. It is highly probable that the results were influenced by the increased prevalence of chronic hepatitis C among patients with HCC, given the negative impact of hepatitis C recurrence on graft survival and mortality before the upcoming of the new direct antivirals [29]. Therefore, the increased mortality rates found in kHCC and iHCC patients may be justified by both, the tumor recurrence, and by the untreated severe hepatitis C recurrence.

The present study is hampered by its retrospective design, unicenter involvement and reduced number of patients in the iHCC subgroup. These limitations may have weakened some analyses, and prevented solid conclusions about the true impact of a closer imaging surveillance on iHCC avoidance and prevention of tumor recurrence. However, the use of prospectively collected data with homogeneous pathological examinations, and the control for possible confounding factors by using multiple Cox’s regression have allowed to obtain sufficiently solid results to aid clinical decisions.

In conclusion, iHCC is found in 8.7% of LT candidates, and shows similar histopathological features and long-term outcome as compared with kHCC. A close protocolized surveillance based on high resolution imaging techniques is highly desirable for patients awaiting LT, particularly in settings with prolonged waiting lists. This strategy would decrease the prevalence of iHCC, thus resulting in a more appropriate selection and prioritization of candidates, and indication of bridging ablative therapies. Specific immunosuppression strategies applied to patients with kHCC (ie. early minimization of calcineurin inhibitors [23] and perhaps combination with mTOR inhibitors [30]) should be also extended to patients with iHCC. The combination of both strategies, aggressive screening and tailored immunosuppression, would reduce the risk of HCC recurrence after LT.

## Supporting information

S1 File Minimum dataset. (SAV)

S2 File Selected results. (SPO)

## Abbreviations

AFP: alpha-fetoprotein
CT: computed tomography
HBV: hepatitis B virus
HCC: hepatocellular carcinoma
HCV: hepatitis C virus
iHCC: incidental hepatocellular carcinoma
IQR: interquartile range
kHCC: previously known hepatocellular carcinoma
LT: liver transplantation
MELD: Model for End-stage Liver Disease
MRI: magnetic resonance imaging
nHCC: neither previously known nor incidental hepatocellular carcinoma
